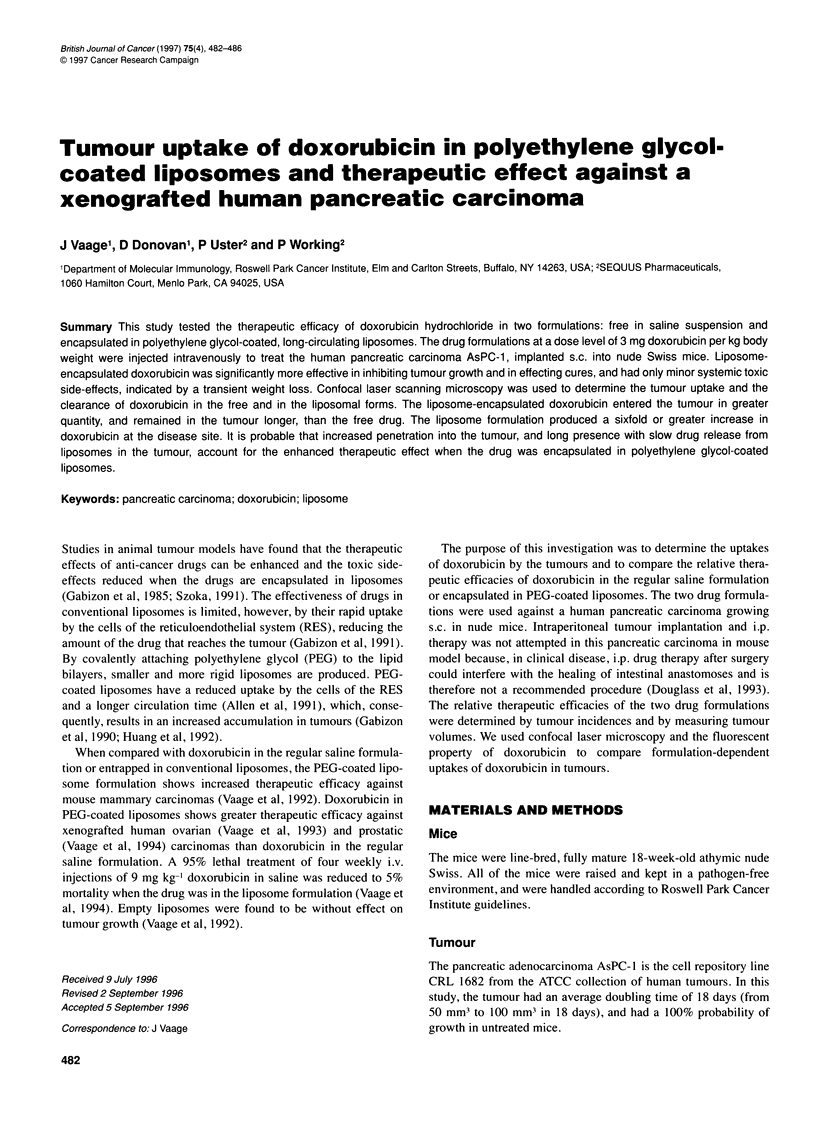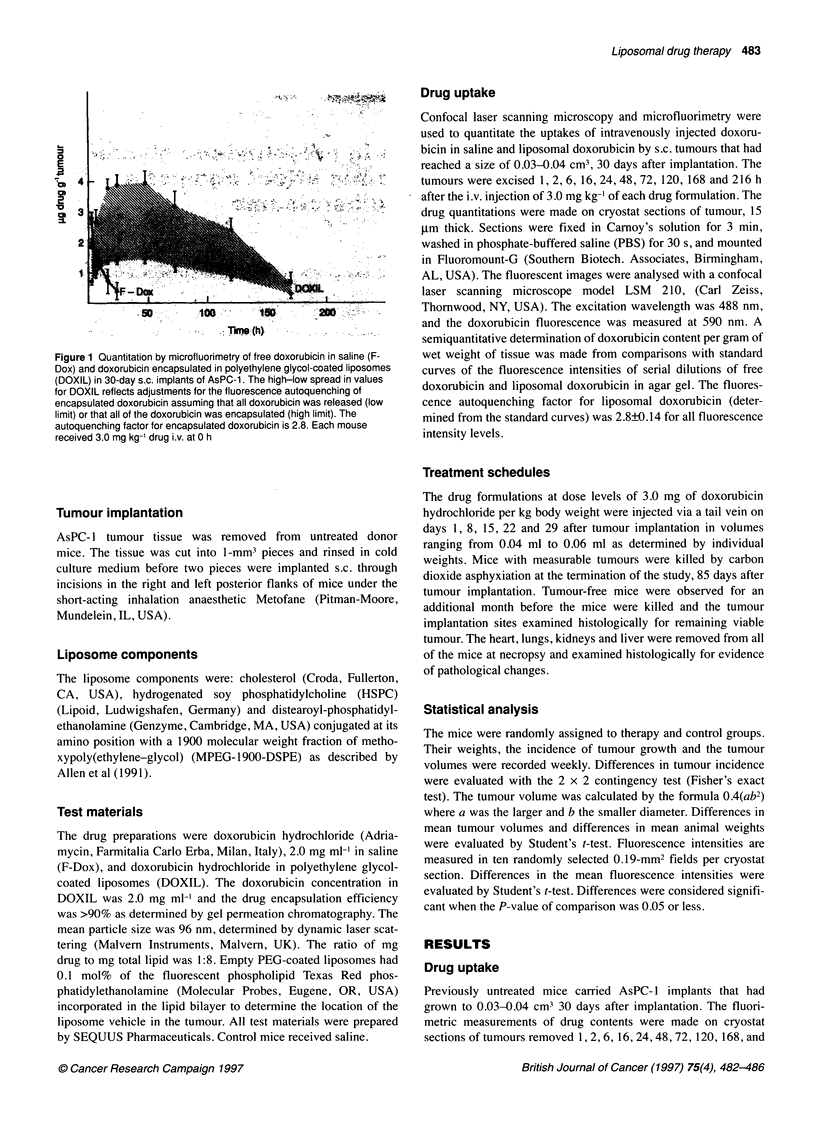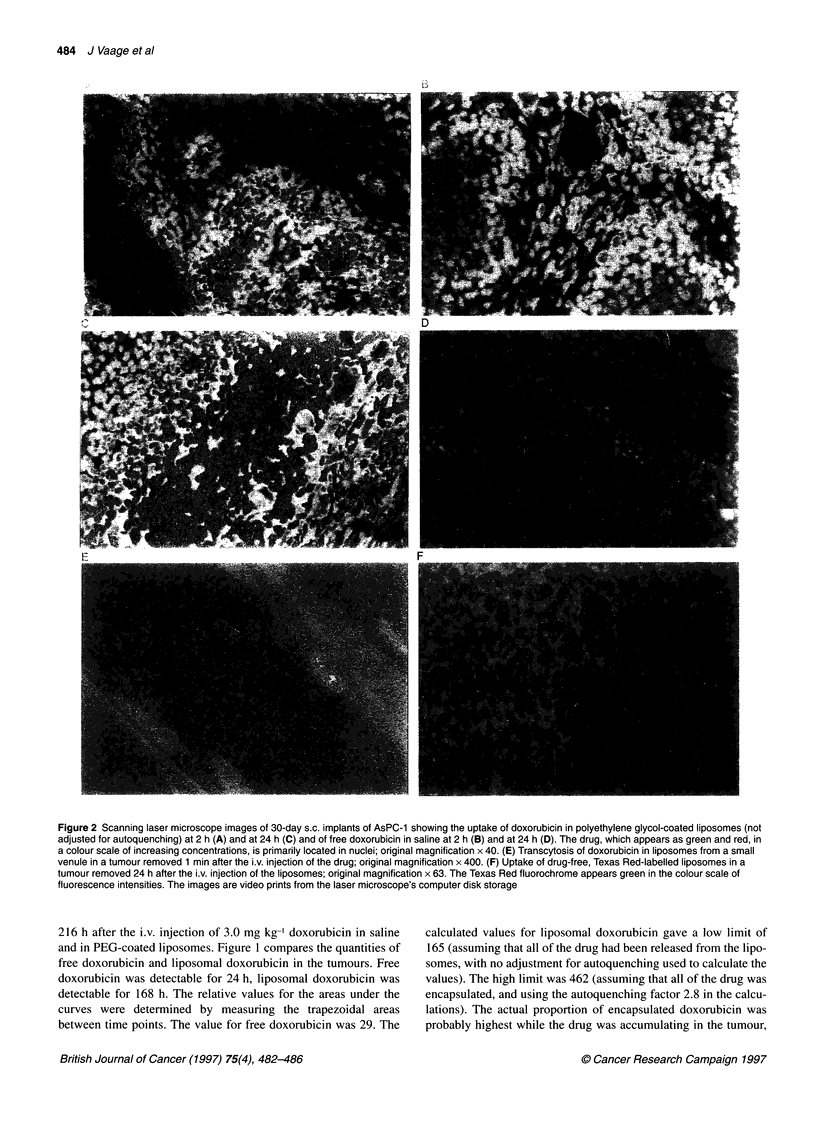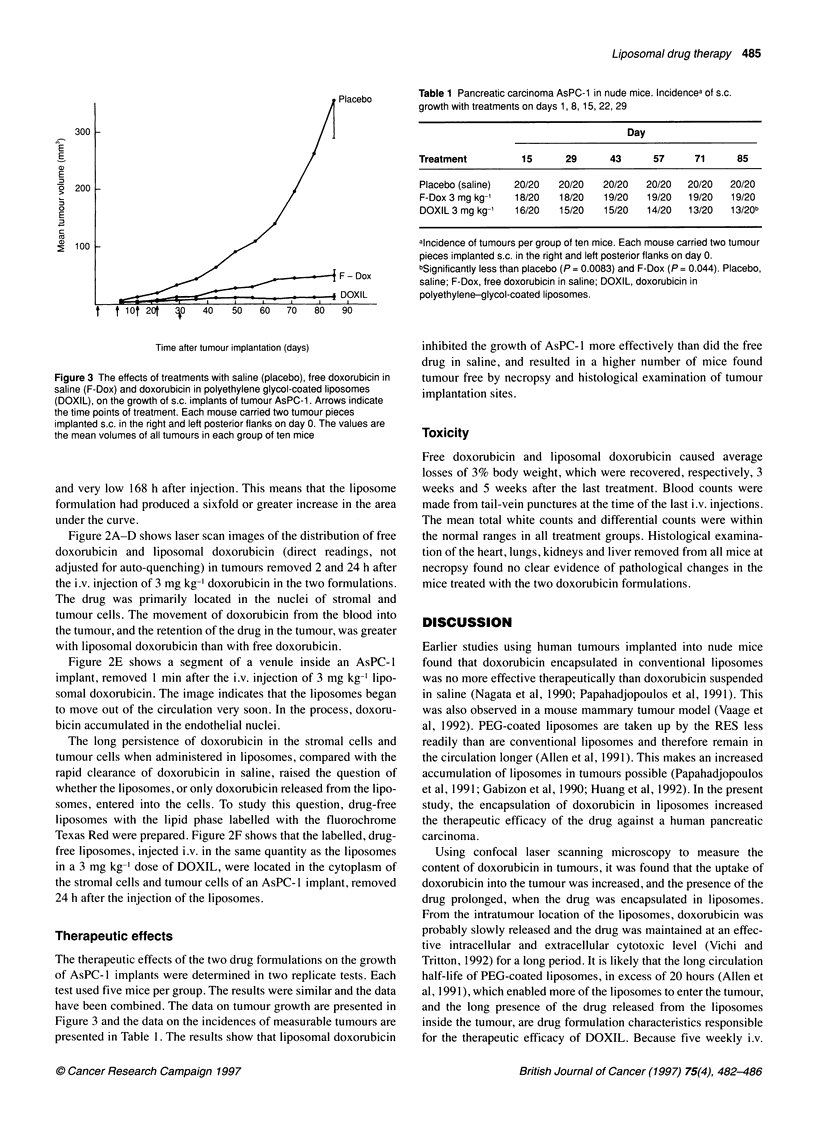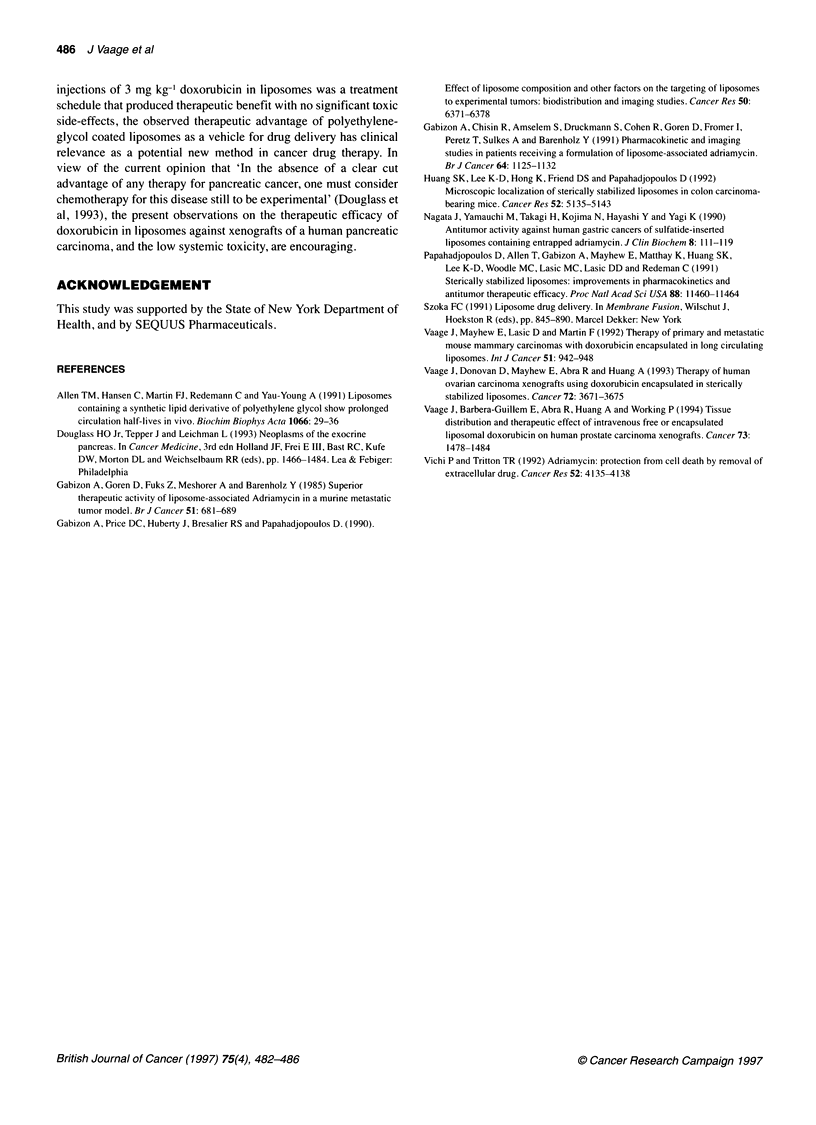# Tumour uptake of doxorubicin in polyethylene glycol-coated liposomes and therapeutic effect against a xenografted human pancreatic carcinoma.

**DOI:** 10.1038/bjc.1997.84

**Published:** 1997

**Authors:** J. Vaage, D. Donovan, P. Uster, P. Working

**Affiliations:** Department of Molecular Immunology, Roswell Park Cancer Institute, Buffalo, NY 14263, USA.

## Abstract

**Images:**


British Journal of Cancer (1997) 75(4), 482–486
© 1997 Cancer Research Campaign

### Tumour uptake of doxorubicin in polyethylene glycol-coated liposomes and therapeutic effect against a xenografted human pancreatic carcinoma

J Vaage[1], D Donovan[1], P Uster[2] and P Working[2]

[1]Department of Molecular Immunology, Roswell Park Cancer Institute, Elm and Carlton Streets, Buffalo, NY 14263, USA; [2]SEQUUS Pharmaceuticals, 1060 Hamilton Court, Menlo Park, CA 94025, USA

**Summary** This study tested the therapeutic efficacy of doxorubicin hydrochloride in two formulations: free in saline suspension and encapsulated in polyethylene glycol-coated, long-circulating liposomes. The drug formulations at a dose level of 3 mg doxorubicin per kg body weight were injected intravenously to treat the human pancreatic carcinoma AsPC-1, implanted s.c. into nude Swiss mice. Liposome-encapsulated doxorubicin was significantly more effective in inhibiting tumour growth and in effecting cures, and had only minor systemic toxic side-effects, indicated by a transient weight loss. Confocal laser scanning microscopy was used to determine the tumour uptake and the clearance of doxorubicin in the free and in the liposomal forms. The liposome-encapsulated doxorubicin entered the tumour in greater quantity, and remained in the tumour longer, than the free drug. The liposome formulation produced a sixfold or greater increase in doxorubicin at the disease site. It is probable that increased penetration into the tumour, and long presence with slow drug release from liposomes in the tumour, account for the enhanced therapeutic effect when the drug was encapsulated in polyethylene glycol-coated liposomes.

**Keywords:** pancreatic carcinoma; doxorubicin; liposome

Studies in animal tumour models have found that the therapeutic effects of anti-cancer drugs can be enhanced and the toxic side-effects reduced when the drugs are encapsulated in liposomes (Gabizon et al, 1985; Szoka, 1991). The effectiveness of drugs in conventional liposomes is limited, however, by their rapid uptake by the cells of the reticuloendothelial system (RES), reducing the amount of the drug that reaches the tumour (Gabizon et al, 1991). By covalently attaching polyethylene glycol (PEG) to the lipid bilayers, smaller and more rigid liposomes are produced. PEG-coated liposomes have a reduced uptake by the cells of the RES and a longer circulation time (Allen et al, 1991), which, consequently, results in an increased accumulation in tumours (Gabizon et al, 1990; Huang et al, 1992).

When compared with doxorubicin in the regular saline formulation or entrapped in conventional liposomes, the PEG-coated liposome formulation shows increased therapeutic efficacy against mouse mammary carcinomas (Vaage et al, 1992). Doxorubicin in PEG-coated liposomes shows greater therapeutic efficacy against xenografted human ovarian (Vaage et al, 1993) and prostatic (Vaage et al, 1994) carcinomas than doxorubicin in the regular saline formulation. A 95% lethal treatment of four weekly i.v. injections of 9 mg $kg^{-1}$ doxorubicin in saline was reduced to 5% mortality when the drug was in the liposome formulation (Vaage et al, 1994). Empty liposomes were found to be without effect on tumour growth (Vaage et al, 1992).

*Received 9 July 1996*
*Revised 2 September 1996*
*Accepted 5 September 1996*

*Correspondence to:* J Vaage

The purpose of this investigation was to determine the uptakes of doxorubicin by the tumours and to compare the relative therapeutic efficacies of doxorubicin in the regular saline formulation or encapsulated in PEG-coated liposomes. The two drug formulations were used against a human pancreatic carcinoma growing s.c. in nude mice. Intraperitoneal tumour implantation and i.p. therapy was not attempted in this pancreatic carcinoma in mouse model because, in clinical disease, i.p. drug therapy after surgery could interfere with the healing of intestinal anastomoses and is therefore not a recommended procedure (Douglass et al, 1993). The relative therapeutic efficacies of the two drug formulations were determined by tumour incidences and by measuring tumour volumes. We used confocal laser microscopy and the fluorescent property of doxorubicin to compare formulation-dependent uptakes of doxorubicin in tumours.

### MATERIALS AND METHODS

### Mice

The mice were line-bred, fully mature 18-week-old athymic nude Swiss. All of the mice were raised and kept in a pathogen-free environment, and were handled according to Roswell Park Cancer Institute guidelines.

### Tumour

The pancreatic adenocarcinoma AsPC-1 is the cell repository line CRL 1682 from the ATCC collection of human tumours. In this study, the tumour had an average doubling time of 18 days (from 50 $mm^3$ to 100 $mm^3$ in 18 days), and had a 100% probability of growth in untreated mice.

**Figure 1** Quantitation by microfluorimetry of free doxorubicin in saline (F-Dox) and doxorubicin encapsulated in polyethylene glycol-coated liposomes (DOXIL) in 30-day s.c. implants of AsPC-1. The high–low spread in values for DOXIL reflects adjustments for the fluorescence autoquenching of encapsulated doxorubicin assuming that all doxorubicin was released (low limit) or that all of the doxorubicin was encapsulated (high limit). The autoquenching factor for encapsulated doxorubicin is 2.8. Each mouse received 3.0 mg $kg^{-1}$ drug i.v. at 0 h

### Tumour implantation

AsPC-1 tumour tissue was removed from untreated donor mice. The tissue was cut into 1-$mm^3$ pieces and rinsed in cold culture medium before two pieces were implanted s.c. through incisions in the right and left posterior flanks of mice under the short-acting inhalation anaesthetic Metofane (Pitman-Moore, Mundelein, IL, USA).

### Liposome components

The liposome components were: cholesterol (Croda, Fullerton, CA, USA), hydrogenated soy phosphatidylcholine (HSPC) (Lipoid, Ludwigshafen, Germany) and distearoyl-phosphatidylethanolamine (Genzyme, Cambridge, MA, USA) conjugated at its amino position with a 1900 molecular weight fraction of methoxypoly(ethylene–glycol) (MPEG-1900-DSPE) as described by Allen et al (1991).

### Test materials

The drug preparations were doxorubicin hydrochloride (Adriamycin, Farmitalia Carlo Erba, Milan, Italy), 2.0 mg $ml^{-1}$ in saline (F-Dox), and doxorubicin hydrochloride in polyethylene glycol-coated liposomes (DOXIL). The doxorubicin concentration in DOXIL was 2.0 mg $ml^{-1}$ and the drug encapsulation efficiency was >90% as determined by gel permeation chromatography. The mean particle size was 96 nm, determined by dynamic laser scattering (Malvern Instruments, Malvern, UK). The ratio of mg drug to mg total lipid was 1:8. Empty PEG-coated liposomes had 0.1 mol% of the fluorescent phospholipid Texas Red phosphatidylethanolamine (Molecular Probes, Eugene, OR, USA) incorporated in the lipid bilayer to determine the location of the liposome vehicle in the tumour. All test materials were prepared by SEQUUS Pharmaceuticals. Control mice received saline.

### Drug uptake

Confocal laser scanning microscopy and microfluorimetry were used to quantitate the uptakes of intravenously injected doxorubicin in saline and liposomal doxorubicin by s.c. tumours that had reached a size of 0.03–0.04 $cm^3$, 30 days after implantation. The tumours were excised 1, 2, 6, 16, 24, 48, 72, 120, 168 and 216 h after the i.v. injection of 3.0 mg $kg^{-1}$ of each drug formulation. The drug quantitations were made on cryostat sections of tumour, 15 μm thick. Sections were fixed in Carnoy's solution for 3 min, washed in phosphate-buffered saline (PBS) for 30 s, and mounted in Fluoromount-G (Southern Biotech. Associates, Birmingham, AL, USA). The fluorescent images were analysed with a confocal laser scanning microscope model LSM 210, (Carl Zeiss, Thornwood, NY, USA). The excitation wavelength was 488 nm, and the doxorubicin fluorescence was measured at 590 nm. A semiquantitative determination of doxorubicin content per gram of wet weight of tissue was made from comparisons with standard curves of the fluorescence intensities of serial dilutions of free doxorubicin and liposomal doxorubicin in agar gel. The fluorescence autoquenching factor for liposomal doxorubicin (determined from the standard curves) was 2.8±0.14 for all fluorescence intensity levels.

### Treatment schedules

The drug formulations at dose levels of 3.0 mg of doxorubicin hydrochloride per kg body weight were injected via a tail vein on days 1, 8, 15, 22 and 29 after tumour implantation in volumes ranging from 0.04 ml to 0.06 ml as determined by individual weights. Mice with measurable tumours were killed by carbon dioxide asphyxiation at the termination of the study, 85 days after tumour implantation. Tumour-free mice were observed for an additional month before the mice were killed and the tumour implantation sites examined histologically for remaining viable tumour. The heart, lungs, kidneys and liver were removed from all of the mice at necropsy and examined histologically for evidence of pathological changes.

### Statistical analysis

The mice were randomly assigned to therapy and control groups. Their weights, the incidence of tumour growth and the tumour volumes were recorded weekly. Differences in tumour incidence were evaluated with the 2 × 2 contingency test (Fisher's exact test). The tumour volume was calculated by the formula $0.4(ab^2)$ where $a$ was the larger and $b$ the smaller diameter. Differences in mean tumour volumes and differences in mean animal weights were evaluated by Student's $t$-test. Fluorescence intensities are measured in ten randomly selected 0.19-$mm^2$ fields per cryostat section. Differences in the mean fluorescence intensities were evaluated by Student's $t$-test. Differences were considered significant when the $P$-value of comparison was 0.05 or less.

### RESULTS

### Drug uptake

Previously untreated mice carried AsPC-1 implants that had grown to 0.03–0.04 $cm^3$ 30 days after implantation. The fluorimetric measurements of drug contents were made on cryostat sections of tumours removed 1, 2, 6, 16, 24, 48, 72, 120, 168, and

© Cancer Research Campaign 1997

**Figure 2** Scanning laser microscope images of 30-day s.c. implants of AsPC-1 showing the uptake of doxorubicin in polyethylene glycol-coated liposomes (not adjusted for autoquenching) at 2 h (**A**) and at 24 h (**C**) and of free doxorubicin in saline at 2 h (**B**) and at 24 h (**D**). The drug, which appears as green and red, in a colour scale of increasing concentrations, is primarily located in nuclei; original magnification × 40. (**E**) Transcytosis of doxorubicin in liposomes from a small venule in a tumour removed 1 min after the i.v. injection of the drug; original magnification × 400. (**F**) Uptake of drug-free, Texas Red-labelled liposomes in a tumour removed 24 h after the i.v. injection of the liposomes; original magnification × 63. The Texas Red fluorochrome appears green in the colour scale of fluorescence intensities. The images are video prints from the laser microscope's computer disk storage

216 h after the i.v. injection of 3.0 mg $kg^{-1}$ doxorubicin in saline and in PEG-coated liposomes. Figure 1 compares the quantities of free doxorubicin and liposomal doxorubicin in the tumours. Free doxorubicin was detectable for 24 h, liposomal doxorubicin was detectable for 168 h. The relative values for the areas under the curves were determined by measuring the trapezoidal areas between time points. The value for free doxorubicin was 29. The calculated values for liposomal doxorubicin gave a low limit of 165 (assuming that all of the drug had been released from the liposomes, with no adjustment for autoquenching used to calculate the values). The high limit was 462 (assuming that all of the drug was encapsulated, and using the autoquenching factor 2.8 in the calculations). The actual proportion of encapsulated doxorubicin was probably highest while the drug was accumulating in the tumour,

© Cancer Research Campaign 1997

**Figure 3** The effects of treatments with saline (placebo), free doxorubicin in saline (F-Dox) and doxorubicin in polyethylene glycol-coated liposomes (DOXIL), on the growth of s.c. implants of tumour AsPC-1. Arrows indicate the time points of treatment. Each mouse carried two tumour pieces implanted s.c. in the right and left posterior flanks on day 0. The values are the mean volumes of all tumours in each group of ten mice and very low 168 h after injection. This means that the liposome formulation had produced a sixfold or greater increase in the area under the curve.

Figure 2A–D shows laser scan images of the distribution of free doxorubicin and liposomal doxorubicin (direct readings, not adjusted for auto-quenching) in tumours removed 2 and 24 h after the i.v. injection of 3 mg $kg^{-1}$ doxorubicin in the two formulations. The drug was primarily located in the nuclei of stromal and tumour cells. The movement of doxorubicin from the blood into the tumour, and the retention of the drug in the tumour, was greater with liposomal doxorubicin than with free doxorubicin.

Figure 2E shows a segment of a venule inside an AsPC-1 implant, removed 1 min after the i.v. injection of 3 mg $kg^{-1}$ liposomal doxorubicin. The image indicates that the liposomes began to move out of the circulation very soon. In the process, doxorubicin accumulated in the endothelial nuclei.

The long persistence of doxorubicin in the stromal cells and tumour cells when administered in liposomes, compared with the rapid clearance of doxorubicin in saline, raised the question of whether the liposomes, or only doxorubicin released from the liposomes, entered into the cells. To study this question, drug-free liposomes with the lipid phase labelled with the fluorochrome Texas Red were prepared. Figure 2F shows that the labelled, drug-free liposomes, injected i.v. in the same quantity as the liposomes in a 3 mg $kg^{-1}$ dose of DOXIL, were located in the cytoplasm of the stromal cells and tumour cells of an AsPC-1 implant, removed 24 h after the injection of the liposomes.

### Therapeutic effects

The therapeutic effects of the two drug formulations on the growth of AsPC-1 implants were determined in two replicate tests. Each test used five mice per group. The results were similar and the data have been combined. The data on tumour growth are presented in Figure 3 and the data on the incidences of measurable tumours are presented in Table 1. The results show that liposomal doxorubicin

**Table 1** Pancreatic carcinoma AsPC-1 in nude mice. Incidence[a] of s.c. growth with treatments on days 1, 8, 15, 22, 29

| | Day | | | | | |
|---|---|---|---|---|---|---|
| Treatment | 15 | 29 | 43 | 57 | 71 | 85 |
| Placebo (saline) | 20/20 | 20/20 | 20/20 | 20/20 | 20/20 | 20/20 |
| F-Dox 3 mg $kg^{-1}$ | 18/20 | 18/20 | 19/20 | 19/20 | 19/20 | 19/20 |
| DOXIL 3 mg $kg^{-1}$ | 16/20 | 15/20 | 15/20 | 14/20 | 13/20 | 13/20[b] |

[a]Incidence of tumours per group of ten mice. Each mouse carried two tumour pieces implanted s.c. in the right and left posterior flanks on day 0. [b]Significantly less than placebo ($P = 0.0083$) and F-Dox ($P = 0.044$). Placebo, saline; F-Dox, free doxorubicin in saline; DOXIL, doxorubicin in polyethylene–glycol-coated liposomes.

inhibited the growth of AsPC-1 more effectively than did the free drug in saline, and resulted in a higher number of mice found tumour free by necropsy and histological examination of tumour implantation sites.

### Toxicity

Free doxorubicin and liposomal doxorubicin caused average losses of 3% body weight, which were recovered, respectively, 3 weeks and 5 weeks after the last treatment. Blood counts were made from tail-vein punctures at the time of the last i.v. injections. The mean total white counts and differential counts were within the normal ranges in all treatment groups. Histological examination of the heart, lungs, kidneys and liver removed from all mice at necropsy found no clear evidence of pathological changes in the mice treated with the two doxorubicin formulations.

### DISCUSSION

Earlier studies using human tumours implanted into nude mice found that doxorubicin encapsulated in conventional liposomes was no more effective therapeutically than doxorubicin suspended in saline (Nagata et al, 1990; Papahadjopoulos et al, 1991). This was also observed in a mouse mammary tumour model (Vaage et al, 1992). PEG-coated liposomes are taken up by the RES less readily than are conventional liposomes and therefore remain in the circulation longer (Allen et al, 1991). This makes an increased accumulation of liposomes in tumours possible (Papahadjopoulos et al, 1991; Gabizon et al, 1990; Huang et al, 1992). In the present study, the encapsulation of doxorubicin in liposomes increased the therapeutic efficacy of the drug against a human pancreatic carcinoma.

Using confocal laser scanning microscopy to measure the content of doxorubicin in tumours, it was found that the uptake of doxorubicin into the tumour was increased, and the presence of the drug prolonged, when the drug was encapsulated in liposomes. From the intratumour location of the liposomes, doxorubicin was probably slowly released and the drug was maintained at an effective intracellular and extracellular cytotoxic level (Vichi and Tritton, 1992) for a long period. It is likely that the long circulation half-life of PEG-coated liposomes, in excess of 20 hours (Allen et al, 1991), which enabled more of the liposomes to enter the tumour, and the long presence of the drug released from the liposomes inside the tumour, are drug formulation characteristics responsible for the therapeutic efficacy of DOXIL. Because five weekly i.v.

injections of 3 mg $kg^{-1}$ doxorubicin in liposomes was a treatment schedule that produced therapeutic benefit with no significant toxic side-effects, the observed therapeutic advantage of polyethylene-glycol coated liposomes as a vehicle for drug delivery has clinical relevance as a potential new method in cancer drug therapy. In view of the current opinion that 'In the absence of a clear cut advantage of any therapy for pancreatic cancer, one must consider chemotherapy for this disease still to be experimental' (Douglass et al, 1993), the present observations on the therapeutic efficacy of doxorubicin in liposomes against xenografts of a human pancreatic carcinoma, and the low systemic toxicity, are encouraging.

### ACKNOWLEDGEMENT

This study was supported by the State of New York Department of Health, and by SEQUUS Pharmaceuticals.

### REFERENCES

Allen TM, Hansen C, Martin FJ, Redemann C and Yau-Young A (1991) Liposomes containing a synthetic lipid derivative of polyethylene glycol show prolonged circulation half-lives in vivo. *Biochim Biophys Acta* **1066**: 29–36

Douglass HO Jr, Tepper J and Leichman L (1993) Neoplasms of the exocrine pancreas. In *Cancer Medicine*, 3rd edn Holland JF, Frei E III, Bast RC, Kufe DW, Morton DL and Weichselbaum RR (eds), pp. 1466–1484. Lea & Febiger: Philadelphia

Gabizon A, Goren D, Fuks Z, Meshorer A and Barenholz Y (1985) Superior therapeutic activity of liposome-associated Adriamycin in a murine metastatic tumor model. *Br J Cancer* **51**: 681–689

Gabizon A, Price DC, Huberty J, Bresalier RS and Papahadjopoulos D. (1990). Effect of liposome composition and other factors on the targeting of liposomes to experimental tumors: biodistribution and imaging studies. *Cancer Res* **50**: 6371–6378

Gabizon A, Chisin R, Amselem S, Druckmann S, Cohen R, Goren D, Fromer I, Peretz T, Sulkes A and Barenholz Y (1991) Pharmacokinetic and imaging studies in patients receiving a formulation of liposome-associated adriamycin. *Br J Cancer* **64**: 1125–1132

Huang SK, Lee K-D, Hong K, Friend DS and Papahadjopoulos D (1992) Microscopic localization of sterically stabilized liposomes in colon carcinoma-bearing mice. *Cancer Res* **52**: 5135–5143

Nagata J, Yamauchi M, Takagi H, Kojima N, Hayashi Y and Yagi K (1990) Antitumor activity against human gastric cancers of sulfatide-inserted liposomes containing entrapped adriamycin. *J Clin Biochem* **8**: 111–119

Papahadjopoulos D, Allen T, Gabizon A, Mayhew E, Matthay K, Huang SK, Lee K-D, Woodle MC, Lasic MC, Lasic DD and Redeman C (1991) Sterically stabilized liposomes: improvements in pharmacokinetics and antitumor therapeutic efficacy. *Proc Natl Acad Sci USA* **88**: 11460–11464

Szoka FC (1991) Liposome drug delivery. In *Membrane Fusion*, Wilschut J, Hoekston R (eds), pp. 845–890. Marcel Dekker: New York

Vaage J, Mayhew E, Lasic D and Martin F (1992) Therapy of primary and metastatic mouse mammary carcinomas with doxorubicin encapsulated in long circulating liposomes. *Int J Cancer* **51**: 942–948

Vaage J, Donovan D, Mayhew E, Abra R and Huang A (1993) Therapy of human ovarian carcinoma xenografts using doxorubicin encapsulated in sterically stabilized liposomes. *Cancer* **72**: 3671–3675

Vaage J, Barbera-Guillem E, Abra R, Huang A and Working P (1994) Tissue distribution and therapeutic effect of intravenous free or encapsulated liposomal doxorubicin on human prostate carcinoma xenografts. *Cancer* **73**: 1478–1484

Vichi P and Tritton TR (1992) Adriamycin: protection from cell death by removal of extracellular drug. *Cancer Res* **52**: 4135–4138

© Cancer Research Campaign 1997